# ABLE: An Activity-Based Level Set Segmentation Algorithm for Two-Photon Calcium Imaging Data

**DOI:** 10.1523/ENEURO.0012-17.2017

**Published:** 2017-10-30

**Authors:** Stephanie Reynolds, Therese Abrahamsson, Renaud Schuck, P. Jesper Sjöström, Simon R. Schultz, Pier Luigi Dragotti

**Affiliations:** 1 Department of Electrical and Electronic Engineering, Imperial College London, South Kensington Campus, London SW7 2AZ, United Kingdom; 2 Centre for Neurotechnology, Imperial College London, South Kensington Campus, London SW7 2AZ, United Kingdom; 3 Centre for Research in Neuroscience, Brain Repair and Integrative Neuroscience Program, Department of Neurology and Neurosurgery, The Research Institute of the McGill University Health Centre, Montréal General Hospital, Montréal, Québec H3G 1A4, Canada; 4 Department of Bioengineering, Imperial College London, South Kensington Campus, London SW7 2AZ, United Kingdom

**Keywords:** active contour, calcium imaging, fluorescence microscopy, level set method, segmentation

## Abstract

We present an algorithm for detecting the location of cells from two-photon calcium imaging data. In our framework, multiple coupled active contours evolve, guided by a model-based cost function, to identify cell boundaries. An active contour seeks to partition a local region into two subregions, a cell interior and exterior, in which all pixels have maximally “similar” time courses. This simple, local model allows contours to be evolved predominantly independently. When contours are sufficiently close, their evolution is coupled, in a manner that permits overlap. We illustrate the ability of the proposed method to demix overlapping cells on real data. The proposed framework is flexible, incorporating no prior information regarding a cell’s morphology or stereotypical temporal activity, which enables the detection of cells with diverse properties. We demonstrate algorithm performance on a challenging mouse *in vitro* dataset, containing synchronously spiking cells, and a manually labelled mouse *in vivo* dataset, on which ABLE (the proposed method) achieves a 67.5% success rate.

## Significance Statement

Two-photon calcium imaging enables the study of brain activity during learning and behavior at single-cell resolution. To decode neuronal spiking activity from the data, algorithms are first required to detect the location of cells in the video. It is still common for scientists to perform this task manually, as the heterogeneity in cell shape and frequency of cellular overlap impede automatic segmentation algorithms. We developed a versatile algorithm based on a popular image segmentation approach (the level set method) and demonstrated its capability to overcome these challenges. We include no assumptions on cell shape or stereotypical temporal activity. This lends our framework the flexibility to be applied to new datasets with minimal adjustment.

## Introduction

Two-photon calcium imaging has enabled the long-term study of neuronal population activity during learning and behavior (Peron et al., 2015b). State of the art genetically encoded calcium indicators have sufficient signal-to-noise ratio (SNR) to resolve single action potentials (Chen et al., 2013). Furthermore, recent developments in microscope design have extended the possible field-of-view in which individual neurons can be resolved to 9.5 mm^2^ (Stirman et al., 2016), and enabled the simultaneous imaging of separate brain areas (Lecoq et al., 2014). However, a comprehensive study of activity in even one brain area can produce terabytes of imaging data (Peron et al., 2015a), which presents a considerable signal processing problem.

To decode spiking activity from imaging data, one must first be able to accurately detect regions of interest (ROIs), which may be cell bodies, neurites or combinations of the two. Heterogeneity in the appearance of ROIs in imaging datasets complicates the detection problem. The calcium indicator used to generate the imaging video affects both a cell’s resting fluorescence and its apparent shape. For example, some genetically encoded indicators are excluded from the nucleus and therefore produce fluorescent “donuts.” Moreover, imaging data are frequently contaminated with measurement noise and movement artefacts. These challenges necessitate flexible, robust detection algorithms with minimal assumptions on the properties of ROIs.

Manual segmentation of calcium imaging datasets is still commonplace. While this allows the use of complex selection criteria, it is neither reproducible nor scalable. To incorporate implicitly a human’s selection criteria, which can be hard to define mathematically, supervised learning from extensive human-annotated data has been implemented (Valmianski et al., 2010; Apthorpe et al., 2016). Other approaches rely on more general cellular properties, such as their expected size and shape (Ohki et al., 2005) and that they represent regions of peak local correlation (Smith and Häusser, 2010; Kaifosh et al., 2014). The latter approaches use lower-dimensional summary statistics of the data, which reduces computational complexity but does not typically allow detection of overlapping regions.

To better discriminate between neighboring cells, some methods make use of the temporal activity profile of imaging data. The (2 + 1)-D imaging video, which consists of two spatial dimensions and one temporal dimension, is often prohibitively large to work on directly. One family of approaches therefore reshapes the (2 + 1)-D imaging video into a 2-D matrix. The resulting matrix admits a decomposition, derived from a generative model of the imaging video, into two matrices, each encoding spatial and temporal information. The spatial and temporal components are estimated using a variety of methods, such as independent component analysis (Mukamel et al., 2009; Schultz et al., 2009) or non-negative matrix factorization (Maruyama et al., 2014). Recent variants extend the video model to incorporate detail on the structure of neuronal intracellular calcium dynamics (Pnevmatikakis et al., 2016) or the neuropil contamination (Pachitariu et al., 2016). By expressing the (2 + 1)-D imaging video as a 2-D matrix, this type of approach can achieve high processing speeds. This does, however, come at the cost of discarded spatial information, which can necessitate postprocessing with morphologic filters (Pachitariu et al., 2016; Pnevmatikakis et al., 2016).

In this article, we propose a method in which cell boundaries are detected by multiple coupled active contours. To evolve an active contour we use the level set method, which is a popular tool in bioimaging due to its topological flexibility (Delgado-Gonzalo et al., 2015). To each active contour, we associate a higher-dimensional function, referred to as the level set function, whose zero level set encodes the contour location. We implicitly evolve an active contour via the level set function. The evolution of the level set function is driven by a local model of the imaging data temporal activity. The data model includes no assumptions on a cell’s morphology or stereotypical temporal activity. Our algorithm is therefore versatile, it can be applied to a variety of data types with minimal adjustment. For convenience, we refer to our method as ABLE (an activity-based level set method). In the following, we describe the method and demonstrate its versatility and robustness on a range of *in vitro* and *in vivo* datasets.

## Materials and Methods

### Estimating the boundary of an isolated cell

**Figure 1.**
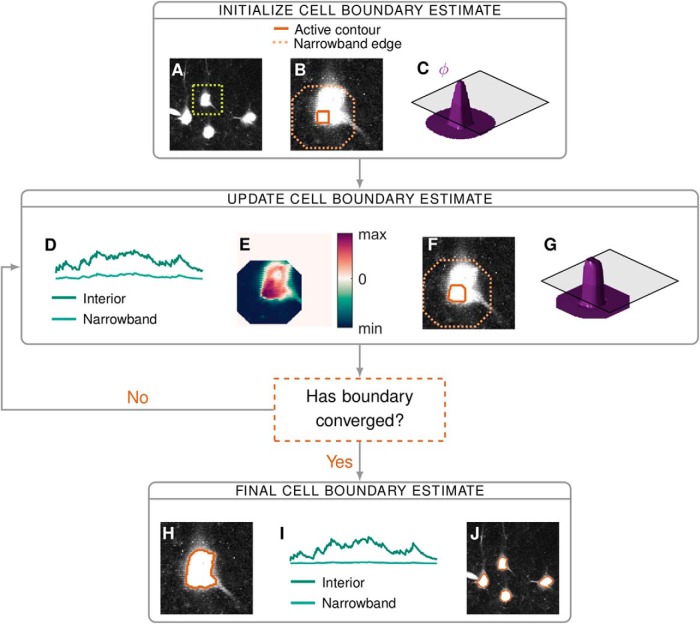
A flow diagram of the main steps of the proposed segmentation algorithm: initialization (***A****–****C***), iterative updates of the estimate (***D–G***), and convergence (***H****-****J***). When cells are sufficiently far apart we can segment them independently, in this example, we focus on the isolated cell in the dashed box on the maximum intensity image in ***A***. We make an initial estimate of the cell interior, from which we form the corresponding narrowband (***B***) and level set function *ϕ* (***C***). Based on the discrepancy between a pixel’s time course and the time courses of the interior and narrowband regions (***D***), we calculate the velocity of *ϕ* at each pixel (***E***). *ϕ* Evolves according to this velocity (***G***), which updates the location of the interior and narrowband (***F***). Final results for: one cell (***H***), the average signals from the corresponding interior and narrowband (***I***), and segmentation of all four cells (***J***).

Consider a small region of a video containing one cell (Fig. 1*A*, inside the dashed box). This region is composed of two subregions: the cell and the background. We want to partition the region into Ω^in^ and Ω^out,^ where Ω^in^ corresponds to the cell and Ω^out^ the background. We compute a feature of the respective subregions, **f**^in^ and **f**^out^, with which to classify pixels into the cell interior or background. In particular, we define $f^{\text{in}}\;\in {\mathbb{R}}^{T}$ and $f^{\text{out}}\;\in {\mathbb{R}}^{T}$ as the average subregion time courses, where *T* is the number of frames in the video. We estimate the optimal partition as the one that minimizes discrepancies between a pixel’s time course and the average time course of the subregion to which it belongs. To calculate this discrepancy, we employ a dissimilarity metric, *D* (see below), which is identically zero when the time courses are perfectly matched and positive otherwise. As such, we minimize the following cost function, which we refer to as the external energy,

(1) $$E_{\text{ext}}(\Omega^{\text{in}}\;,\Omega^{\text{out}}\;)=\int_{\Omega^{\text{in}}\;}D(I(x),\;f^{\text{in}}\;)\text{d}x+\int_{\Omega^{\text{out}}\;}D(I(x),\;f^{\text{out}}\;)\text{d}x,$$

where $I(x)\in {\mathbb{R}}^{T}$ is the time course of pixel **x**.

The cell location estimate is iteratively updated by the algorithm. At each iteration, the cell exterior is defined as the set of pixels within a fixed distance of the current estimate of the cell interior (Fig. 1*B*). The default distance is taken to be two times the expected radius of a cell. We refer to this exterior region as the narrowband to emphasise its proximity to the contour of interest. The boundary between the interior and the narrowband is the active contour. As an active contour is updated, so is the corresponding narrowband (Fig. 1*F*). The region of the video for which the optimal partition is sought is therefore not static; rather, it evolves as an active contour evolves.

### Computing the dissimilarity metric

Because of the heterogeneity of calcium imaging data, we do not use a universal dissimilarity metric. When both the pattern and magnitude of a pixel’s temporal activity are informative, as is typically the case for synthetic dyes, we use a measure based on the Euclidean distance, where

(2) $$D^{E}(I(x),f)=\|I(x)-f{\|}^{2},$$

for $f\in {\mathbb{R}}^{T}$. When we have an image not a video (i.e. **I**(**x**) and **f** are one-dimensional) this dissimilarity metric reduces to the fitting term introduced by Chan and Vese (2001). For datasets in which the fluorescence expression level varies significantly throughout cells and, as a consequence, pixels in the same cell exhibit the same pattern of activity at different magnitudes, we use a measure based on the correlation, such that

(3) $$D^{C}(I(x),f)=1-\text{corr}(I(x),f),$$

where corr represents the Pearson correlation coefficient. In this article, as default, we use the Euclidean dissimilarity metric. Additionally, we present two notable examples in which the correlation-based metric is preferable.

### External energy for neighboring cells

We now extend the cost function presented in Equation 1 to one suitable for partitioning a region into multiple cell interiors, {Ω^in,1^, Ω^in,2^,…, Ω^in,^*^M^*}, and a global exterior, Ω^out,^ which encompasses the narrowbands of all the cells. We denote with **f**^in,^*^i^* the average time course of pixels exclusively in Ω^in,^*^i^*. Due to the relatively low axial resolution of a two-photon microscope, fluorescence intensity at one pixel can originate from multiple cells in neighboring z-planes. Accordingly, we allow cell interiors to overlap when this best fits the data. In particular, we assume that a pixel in multiple cells would have a time course well fit by the sum of the interior time courses for each cell. The external energy in the case of multiple cells is thus

(4) $$E_{\text{ext}}(\Omega^{\text{in},1}\;,\ldots ,\Omega^{\text{in},M}\;,\Omega^{\text{out}}\;)=\underset{\Omega^{\text{out}}\;}{\int }D(I(x),\;f^{\text{out}}\;)\text{d}x\;\;\;\;\;\;\;\;\;\;+\underset{\text{inside}}{\int }D(I(x),\;\underset{i\in C(x)}{\sum }f^{\text{in},i}\;)\text{d}x,$$

where the area termed “inside” denotes the union of all cell interiors and the function *C*(**x**) identifies all cells whose interior contains pixel **x**. When the region to be partitioned contains only one cell, the external energy in Equation 4 reduces to that in Equation 1.

### Level set method

It is not possible to find an optimal cell boundary by minimizing the external energy directly (Chan and Vese, 2001). An alternative solution is to start from an initial estimate, see below, and evolve this estimate in terms of an evolution parameter *τ*. In this approach, the boundary is called an active contour. To update the active contour we use the level set method of Osher and Sethian (1988). This method was first introduced to image processing by Caselles et al. (1993) and Malladi et al. (1995); it has since found widespread use in the field. We implicitly represent the evolving boundary estimate of the *i*^th^ cell, the *i*^th^ active contour, by a function *ϕ_i_*, where *ϕ_i_* is positive for all pixels in the cell interior, negative for those in the narrowband and zero for all pixels on the boundary (Fig. 1*C*). We refer to *ϕ_i_* as a level set function, as its zero level set identifies the contour of interest. We note that since the contour evolves with *τ*, *ϕ_i_* itself depends on *τ*. In the following, we present a set of *M* partial differential equations (PDEs), one for each active contour, derived in part from Equation 4, which dictate the evolution of the level set functions. The solution to the set of PDEs yields (as the zero level sets) the cell boundaries which minimize the external energy in Equation 4.

From the external energy and a regularization term (Li et al., 2010), we define a new cost function

(5) $$E(\phi_{1},\ldots ,\phi_{M})=\lambda E_{\text{ext}}(\phi_{1},\ldots ,\phi_{M})+\mu R(\phi_{1},\ldots ,\phi_{M}),$$

where the arguments to the external energy in Equation 4 are replaced by the corresponding level set functions. The parameters *λ* and *μ* are real-valued scalars, which define the relative weight of the external energy and the regularizer. The regularizer is designed to ensure that a level set function varies smoothly in the vicinity of its active contour. The corresponding regularization energy is minimized when *ϕ_i_* has gradient of magnitude one near the active contour and magnitude zero far from the contour. An example of such a function, a signed distance function (which is the shape of all level set functions on initialization), can be seen in Figure 1*C*.

A standard way to obtain the level set function that minimizes the cost function is to find the steady-state solution to the gradient flow equation (Aubert and Kornprobst, 2006), we do this for each *ϕ_i_*:

(6) $$\frac{\partial \phi_{i}}{\partial \tau }=-\frac{\partial E}{\partial \phi_{i}},$$

for *i* ∈ {1,2,…,M}. From Equation 5 we obtain

(7) $$\frac{\partial \phi_{i}}{\partial \tau }=-\left(\lambda \frac{\partial E_{\text{ext}}}{\partial \phi_{i}}+\mu \frac{\partial R}{\partial \phi_{i}}\right).$$

We solve this PDE numerically, by discretizing the evolution parameter *τ*, such that

(8) $$\phi_{i}(\tau +1)=\phi_{i}(\tau )-\Delta \tau \left(\lambda \frac{\partial E_{\text{ext}}}{\partial \phi_{i}}+\mu \frac{\partial R}{\partial \phi_{i}}\right).$$

The regularization term, which encourages *ϕ_i_* to vary smoothly in the image plane, helps to ensure the accurate computation of the numerical solution.

At every timestep *τ*, each level set function is consecutively updated until convergence. We must retain μΔτ<0.25 to satisfy the Courant-Friedrichs-Lewy condition (Li et al., 2010), a necessary condition for the convergence of a numerically-solved PDE. This condition requires that the numerical waves propagate at least as fast as the physical waves (Osher and Fedkiw, 2003). We therefore set Δ *τ* = 10 and *μ* = 0.2/Δ *τ*. For each dataset, we tune the value of *λ* based on the algorithm performance on a small section of the video. To attain segmentation results on the real datasets presented in this article, we use *λ* = 150 (see Results, ABLE is robust to heterogeneity in cell shape and baseline intensity), *λ* = 50 (see Results, ABLE detects synchronously spiking, densely packed cells), *λ* = 25 (see Results, Algorithm comparison on manually labeled dataset), and *λ* = 10 (see Results, Spikes are detected from ABLE-extracted time courses with high temporal precision).

### External velocity

The movement of a level set function, *ϕ_i_*, is driven by the derivatives in Equation 8, $\partial E_{\text{ext}}/\partial \phi_{i}$ provides the impetus from the video data and $\partial R/\partial \phi_{i}$ the impetus from the regularizer. In the following, we outline the calculation and interpretation of $\partial E_{\text{ext}}/\partial \phi_{i}$; the regularizer is standard and its derivative is detailed in Li et al. (2010). As is typical in the level set literature (Zhao et al., 1996; Li et al., 2010), using an approximation of the Dirac delta function $\delta_{\epsilon }$, we obtain an approximation of the derivative: $\partial E_{\text{ext}}/\partial \phi_{i}(x)=\delta_{\epsilon }\left(\phi_{i}(x)\right)V_{i}(x)$, where

(9) $$V_{i}(x)=\{\begin{array}{ll} D(I(x),\;f^{\text{in},i}\;)-D(I(x),\;f^{\text{out}}\;)\; & x\;\text{not in neighboring cell,}\\ D(I(x),\;f^{\text{in},i}\;+\underset{j\in C(x)}{\sum }f^{\text{in},j}\;)-D(I(x),\;\underset{j\in C(x)}{\sum }f^{\text{in},j}\;) & \text{otherwise}. \end{array}$$

We refer to *V_i_*(**x**) as the external velocity as it encapsulates the impetus to movement derived from the external energy in Equation 4, see Figure 1*E* for an illustrative example.

The term *δ_ϵ_*, which is only non-zero at pixels on or near the cell boundary, acts as a localization operator, ensuring that the velocity only impacts *ϕ_i_* at pixels in the vicinity of the active contour. The parameter *ϵ* defines the approximate radius, in pixels, of the non-zero band, here, we take *ϵ* = 2. The product with the localization operator means that, in practice, the external velocity must only be evaluated at pixels on or near the cell boundary. As a consequence, although the external velocity contains contributions from all cells in the video, the problem remains local, only neighboring cells directly affect a cell’s evolution.

**Figure 2.**
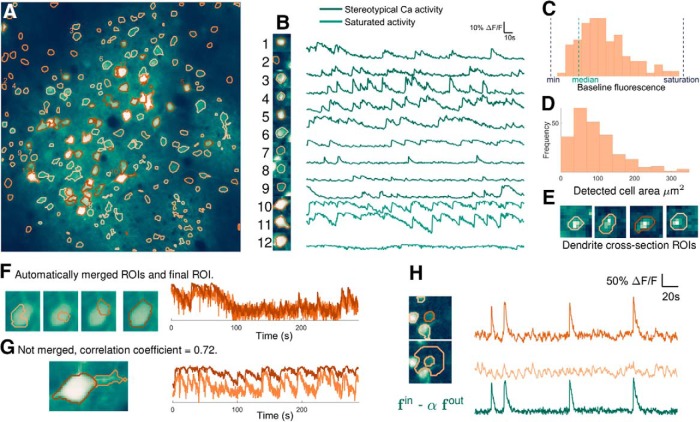
ABLE detects cells with varying size, shape, and baseline intensity from mouse *in vivo* imaging data. The 236 detected ROIs are superimposed on the mean image of the imaging video (***A***). Extracted neuropil-corrected time series and corresponding ROIs are displayed for a subset of the detected regions (***B***). Cells with both stereotypical calcium transient activity (***B***, 1–9) and saturating fluorescence (***B***, 10–12) are detected. The performance of ABLE does not deteriorate due to intensity inhomogeneity: ROIs with baseline fluorescence from beneath the video median to just below saturation are detected (***C***). The area of detected regions varies (***D***) with the smallest ROIs corresponding to cross-sections of dendrites (***E***). Neighboring regions with sufficiently high correlation are merged (***F***), those with lower correlation are not merged (***G***). In ***F***, we plot the ROIs before and after merging along with the corresponding neuropil-corrected time courses. In ***G***, we plot the separate ROIs and the neuropil-corrected time courses. The proposed method naturally facilitates neuropil-correction, the removal of the weighted, local neuropil time course from the raw cellular time course (***H***).

Although Ω^out^ represents a global exterior, in practice, we calculate the corresponding time course in Equation 9, **f**^out^, locally. To evaluate the external velocity of an active contour, we calculate **f**^out^ as the average time course from pixels in the corresponding narrowband. This allows us to neglect components such as intensity inhomogeneity and neuropil contamination (Fig. 2), which we assume vary on a scale larger than that of the narrowband.

The external velocity of a single active contour (Eq. 9) can be interpreted as follows: if a pixel, not in another cell, has time course more similar to that of the contour interior than the narrowband, then the contour moves to incorporate that pixel. If a pixel in another cell has time course better matched by the sum of the interior time courses of cells containing that pixel plus the interior time course of the evolving active contour, then the contour moves to incorporate it. Otherwise, the contour is repelled from that pixel.

### Initialization

We devised an automatic initialization algorithm which selects connected areas of either peak local correlation or peak mean intensity as initial ROI estimates. Initializing areas of peak mean intensity, which may correspond to artefacts rather than active cells (see, e.g., the electrode in Fig. 1*A*), is essential so that these regions do not distort the narrowband signal of another ROI. We first compute the correlation image of the video. For each pixel, this is the average correlation between that pixel’s time course and those of the pixels in its 8-connected neighborhood. Local peaks in this image and the mean intensity image are identified (by a built-in MATLAB function, “imextendedmax”) as candidate ROIs. The selectivity of the initialization is set by a tuning parameter *α*, which defines the relative height with respect to neighboring pixels (in units of SD of the input image) of the peaks that are suppressed. The higher the value of *α*, the more conservative the initialization. We have found it best to use a low value for *α* (in the range 0.2-0.8) so as to overestimate the number of ROIs; redundant estimates are automatically pruned during the update phase of the algorithm. Moreover, smaller values of *α* produce smaller initializations, which reduce errors due to initializations composed of multiple cells.

### Convergence

We stop updating a contour estimate if a maximum number of iterations *N*_max_ has been reached or the active contour has converged, using one or both of these conditions is common in the active contour literature (Li et al., 2010; Delgado-Gonzalo and Unser, 2013). A contour is deemed to have converged if, in *N*_con_ consecutive iterations, the number of pixels that are added to or removed from the interior is less than *ρ*. As default, we take *N*_max_ = 100, *N*_con_ = 40 and *ρ* = 2.

The complexity of the level set method is intrinsically related to the dimensionality of the active contour; the number of frames of the video is only relevant to the evaluation of the external velocity (Eq. 9), which accounts for a small fraction of the computational cost. In Table 1, we demonstrate that increasing video length by a factor of 10 has only a minor impact on processing time. As the framework includes no assumptions on an ROI’s stereotypical temporal activity, before segmentation a video can be downsampled by averaging consecutive samples, thereby simultaneously enabling the processing of longer videos and increasing SNR.

**Table 1.** Runtime (minutes) on synthetic data of size 512 × 512 × T

		Number of cells		
		25	125	225
Number of frames (*T*)	100	1.1	6.5	11.2
	1000	1.3	6.5	12.7

On synthetic data with dimensions 512 × 512 × *T*, the runtime of ABLE (minutes) increases linearly with the number of cells and is not significantly affected by increasing number of frames, *T*. Runtime was measured on a PC with 3.4 GHz Intel Core i7 CPU.

Increasing cell density principally impacts the calculation of the external velocity and does, therefore, not alter the computational complexity of the algorithm. On synthetic data, we observe that increasing cell density only marginally affects the convergence rate (Table 2). As emphasized above (see External velocity), updating an active contour is a local problem, consequently, we observe that algorithm runtime increases linearly with the total number of cells (Table 1). Due to the independence of spatially separate ROIs in our framework, further performance speed-ups are achievable by parallelizing the computation.

**Table 2.** Number of iterations to convergence as cell density increases

Number of neighbors	0	1	2	3	4
Number of iterations	33	33	35	35	36

On synthetic data the average number of iterations to convergence, over 100 realizations of noisy data, marginally increases as the number of cells in a given cell’s narrowband (“neighboring cells”) increases.

### Merging and pruning ROIs

ABLE automatically merges two cells if they are sufficiently close and their interiors sufficiently correlated, a strategy previously employed in the constrained matrix factorization algorithm of Pnevmatikakis et al. (2016). When two contours are merged, their respective level set functions are replaced with a single level set function, initialized as a signed distance function (Fig. 1*C*), with a zero level set that represents the union of the contour interiors.

The required proximity for two cells to be merged is one cell radius (the expected cell radius is one of two required user input parameters). To determine the correlation threshold, we consider the correlation of two noisy time courses corresponding to the average signals from two distinct sets of pixels belonging to the same cell. We assume the underlying signal components, which correspond to the cellular signal plus background contributions, have maximal correlation but that the additive noise reduces the correlation of the noisy time courses. Assuming the noise processes are independent from the underlying cellular signal and each other, the correlation coefficient of the noisy time courses is

(10) $$\frac{1}{1+10^{-{\text{SNR}}_{\text{dB}}/10}},$$

where SNR_dB_ is the signal-to-noise-ratio (dB) of the noisy time courses. We thus merge components with correlation above this threshold. We select a default correlation threshold of 0.8, derived from a default expected SNR of 5 (dB). The user has the option to input an empirically measured SNR, which updates the correlation threshold using the formula in Equation 10.

A contour is automatically removed (“pruned”) during the update phase if its area is smaller or greater than adjustable minimum or maximum size thresholds, which, as default, are set at 3 and 3*πr*^2^ pixels, respectively, where *r* is the expected radius of a cell.

### Metric definitions

The SNR is defined as the ratio of the power of a signal $\sigma_{x}^{2}$ and the power of the noise $\sigma_{\epsilon }^{2}$, such that ${\text{SNR}}_{\text{pow}}=\sigma_{x}^{2}/\sigma_{\epsilon }^{2}$. We write the SNR in decibels (dB) as ${\text{SNR}}_{\text{dB}}=10\log_{10}\left({\text{SNR}}_{\text{pow}}\right)$.

Given two sets of objects, a ground truth set and a set of estimates, the precision is the percentage of estimates that are also in the ground truth set and the recall is the percentage of ground truth objects that are found in the set of estimates. As a complement of the precision we use the fall-out rate, the percentage of estimates not found in the ground truth set. The success rate (%) is

(11) $$2\;\frac{\text{precision}\;*\;\text{recall}}{\text{precision}+\text{recall}}.$$

When the objects are cells, an estimate is deemed to match a ground truth cell if their centres are within 5 pixels of one another. When the objects are spikes, the required distance is 0.22s (three-sample widths). To quantify spike detection performance, we also use the root-mean-square error, which is the square root of the average squared error between an estimated spike time (${\hat{t}}_{k}\in \mathbb{R}$) and the ground truth spike time ($t_{k}\in \mathbb{R}$).

### Simulations

To quantify segmentation performance, we simulated calcium imaging videos. In the following, we detail the method used to generate the videos. Cellular spike trains are generated from mutually independent Poisson processes. A cell’s temporal activity is the sum of a stationary baseline component, the value of which is selected from a uniform distribution, and a spike train convolved with a stereotypical calcium transient pulse shape. Cells are donut (annulus) shaped to mimic videos generated by genetically encoded calcium indicators, which are excluded from the nucleus. To achieve this, the temporal activity of a pixel in a cell is generated by multiplying the cellular temporal activity vector by a factor in [0,1] that decreases as pixels are further from the cell boundary. When two cells overlap in one pixel, we sum the contributions of both cells at that pixel. Spatially and temporally varying background activity, generated independently from the cellular spiking activity, is present in pixels that do not belong to a cell.

### Software accessibility

The software described in the paper is freely available online at http://github.com/StephanieRey.

### Two-photon calcium imaging of quadruple whole-cell recordings

All procedures conformed to the standards and guidelines set in place by the Canadian Council on Animal Care, under the Research Institute of the McGill University Health Centre animal use protocol number 2011-6041 to PJS. P11-P15 mice of either sex were anaesthetized with isoflurane, decapitated, and the brain was rapidly dissected in 4^∘^C external solution consisting of 125 mM NaCl, 2.5 mM KCl, 1 mM MgCl_2_, 1.25 mM NaH_2_PO_4_, 2 mM CaCl_2_, 26 mM NaHCO_3_, and 25 mM dextrose, bubbled with 95% O_2_/5% CO_2_ for oxygenation and pH. Quadruple whole-cell recordings in acute visual cortex slices were conducted at 32-34°C with internal solution consisting of 5 mM KCl, 115 mM K-gluconate, 10 mM K-HEPES, 4 mM MgATP, 0.3 mM NaGTP, 10 mM Na-phosphocreatine, and 0.1% w/v biocytin, adjusted with KOH to pH 7.2-7.4. On the day of the experiment, 20 μM Alexa Fluor 594 and 180 μM Fluo-5F pentapotassium salt (Life Technologies) were added to the internal solution. Electrophysiology amplifier (Dagan BVC-700A) signals were recorded with a National Instruments PCI-6229 board, using in-house software running in Igor Pro 6 (WaveMetrics). The two-photon imaging workstation was custom built as previously described (Buchanan et al., 2012). Briefly, two-photon excitation was achieved by raster-scanning a Spectraphysics MaiTai BB Ti:Sa laser tuned to 820 nm across the sample using an Olympus 40× objective and galvanometric mirrors (Cambridge Technologies 6215H, 3 mm, 1 ms/line, 256 lines). Substage photomultiplier tube signals (R3896, Hamatsu) were acquired with a National Instruments PCI-6110 board using ScanImage 3.7 running in MATLAB (MathWorks). Layer-5 pyramidal cells were identified by their prominent apical dendrites using infrared video Dodt contrast. Unless otherwise stated, all drugs were obtained from Sigma-Aldrich.

### Two-photon calcium imaging of bulk loaded hippocampal slices

All procedures were performed in accordance with national and institutional guidelines and were approved by the UK Home Office under Project License 70/7355 to SRS. Juvenile wild-type mice of either sex (C57Bl6, P13-P21) were anaesthetized using isoflurane before decapitation procedure. Brain slices (400 μm thick) were horizontally cut in 1-4°C ventilated (95% O_2_, 5% CO_2_) slicing artificial CSF (ACSF: 0.5 mM CaCl_2_, 3.0 mM KCl, 26 mM NaHCO_3_, 1 mM NaH_2_PO_4_, 3.5 mM MgSO_4_, 123 mM sucrose, and 10 mM D-glucose). Hippocampal slices containing dentate gyrus, CA3 and CA1 were taken and resting in ventilated recovery ACSF (rACSF; 2 mM CaCl_2_, 123 mM NaCl, 3.0 mM KCl, 26 mM NaHCO_3_, 1 mM NaH_2_PO_4_, 2 mM MgSO_4_, and 10 mM D-glucose) for 30 min at 37°C. After this, the slices were placed in an incubation chamber containing 2.5 ml of ventilated rACSF and “painted” with 10 μl of the following solution: 50 μg of Cal-520 AM (AAT Bioquest), 2 μl of Pluronic-F127 20% in DMSO (Life Technologies), and 48 μl of DMSO (Sigma Aldrich) where they were left for 30 min at 37^∘^C in the dark. Slices were then washed in rACSF at room temperature for 30 min before imaging. Dentate gyrus granular cells were identified using oblique illumination before being imaged using a standard commercial galvanometric scanner based two-photon microscope (Scientifica) coupled to a mode-locked Mai Tai HP Ti Sapphire (Spectra-Physics) laser system operating at 810 nm. Functional calcium images of granular cells were acquired with a 40× objective (Olympus) by raster scanning a 180 × 180 μm^2^ square field of view at 10 Hz. Electrical stimulation was accomplished with a tungsten bipolar concentric microelectrode (WPI) where the tip of the electrode was placed into the molecular layer of the dentate gyrus (20 pulses with a pulse width of 400 μs and a 60-μA amplitude were delivered into the tissue with a pulse repetition rate of 10 Hz, repeated every 40 s). Unless otherwise stated, all drugs were obtained from Sigma-Aldrich.

## Results

### ABLE is robust to heterogeneity in cell shape and baseline intensity

ABLE detected 236 ROIs with diverse properties from the publicly available mouse *in vivo* imaging dataset of Peron et al. (2015c), see Fig. 2. Automatic initialization on this dataset produced 253 ROIs with 17 automatically removed during the update phase of the algorithm after merging with another region.

To maintain a versatile framework, we included no priors on cellular morphology in the cost function that drives the evolution of an active contour. This allowed ABLE to detect ROIs with varied shapes (Fig. 2*A*) and sizes (Fig. 2*D*). The smaller detected ROIs correspond to cross-sections of dendrites (Fig. 2*E*), whereas the majority correspond to cell bodies. The topological flexibility of the level set method allows cell bodies and neurites to be segmented as separate (Fig. 2*G*) or connected (Fig. 2*A*) objects, depending on the correlation between their time courses. ABLE automatically merges neighboring regions that are sufficiently correlated (Fig. 2*F*). Cell bodies and dendrites that are initialized separately and exhibit distinct temporal activity, however, are not merged. For example, the cell body and neurite in Figure 2*G* were not merged as the cell body’s saturating fluorescence time course was not sufficiently highly correlated with that of the neurite.

Evaluating the external velocity, which drives an active contour’s evolution, requires only data from pixels in close proximity to the contour (see Materials and Methods, External velocity). This region has radius of the same order as that of a cell body. Background intensity inhomogeneity, caused by uneven loading of synthetic dyes or uneven expression of virally inserted genetically encoded indicators, tend to occur on a scale larger than this. On this dataset we show that, as a result of this local approach, ABLE is robust to background intensity inhomogeneity. This is illustrated by the wide range of baseline intensities of the detected ROIs (Fig. 2*C*), some of which are even lower than the video median.

No prior information on stereotypical neuronal temporal activity is included in our framework. Cells detected by ABLE exhibit both stereotypical calcium transient activity (Fig. 2*B*, 1-9) and nonstereotypical activity (Fig. 2*B*, 10-12), perhaps corresponding to saturating fluorescence, higher firing cell types such as interneurons, or non-neuronal cells.

The scattering of photons when imaging at depth can result in leakage of neuropil signal into cellular signal. To obtain decontaminated cellular time courses it is thus important to perform neuropil correction in a subsequent stage, once cells have been located. This involves computation of the decontaminated cellular signal by subtracting the weighted local neuropil signal from the raw cellular signal. As illustrated in Figure 2*H*, the proposed method naturally facilitates neuropil correction, as it computes the required components as a by-product of the segmentation process (see Materials and Methods, External velocity). The appropriate value of the weight parameter varies depending on the imaging set-up (Kerlin et al., 2010; Chen et al., 2013; Peron et al., 2015a). We therefore do not include neuropil-correction as a stage of the algorithm, preferring instead to allow users the flexibility to choose the appropriate parameter in postprocessing.

### ABLE demixes overlapping cells

**Figure 3.**
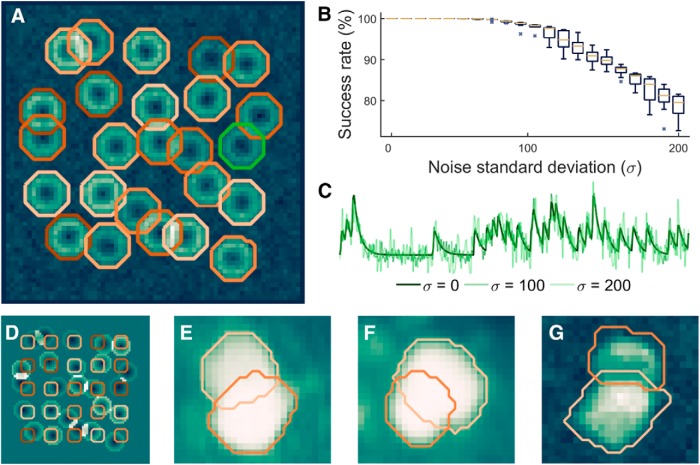
ABLE demixes overlapping cells in real and simulated data. With high accuracy, we detect the true boundaries of overlapping cells from noisy simulated data, the detected contours for one realization of noise with SD (*σ*) 60 are plotted on the correlation image in ***A***. Given an initialization on a fixed grid, displayed on the mean image in ***D***, we detect the true cell boundaries with success rate of at least 99% for *σ* < 90 (***B***). The central marker and box edges in ***B*** indicate the median and the 25th and 75th percentiles, respectively. For noise level reference, we plot the average time course from inside the green contour in ***A*** at various levels (***C***). ABLE demixes overlapping cells in real GCaMP6s mouse *in vivo* data, detected boundaries are superimposed on the mean image (***E***, ***F***) and correlation image (***G***), respectively.

When imaging through scattering tissue, a two-photon microscope can have relatively low axial resolution (on the order of ten microns) in comparison to its excellent lateral resolution. As a consequence, the photons collected at one pixel can in some cases originate from multiple cells in a range of z-planes. For this reason, cells can appear to overlap in an imaging video (Fig. 3*E*). It is crucial that segmentation algorithms can delineate the true boundary of “overlapping” cells, which we refer to as “demixing,” so that the functional activity of each cell can be correctly extracted and analyzed. In a set of experiments on real and simulated data, we demonstrated that ABLE can demix overlapping cells.

On synthetic data containing 25 cells, 17 of which had some overlap with another cell, we measured the success rate of ABLE’s segmentation compared to the ground truth cell locations (Fig. 3*A-C*), when the algorithm was initialized on a fixed grid (Fig. 3*D*). For full description of the performance metric used, see Materials and Methods, Metric definitions. Performance was measured over 10 realizations of noise at each noise level. On average, over all cells and noise realizations, ABLE achieved success rate >99% when the noise SD was <90 (Fig. 3*B*). Cells were simulated with uneven brightness to mimic the donut cells generated by some genetically encoded indicators that are excluded from the nucleus. Consequently, the correlation-based dissimilarity metric was used on this data. As a result, pixels with significantly different resting fluorescence, but identical temporal activity pattern, were segmented in the same cell (Fig. 3*A*).

On the publicly available mouse *in vivo* imaging dataset of Peron et al. (2015c), ABLE demixed overlapping cells (Fig. 3*E*,*F*). In this dataset, the vibrissal cortex was imaged at various depths, from layer 1 to deep layer 3, while the mouse performed a pole localization task (Guo et al., 2014; Peron et al., 2015a). Some cells appear to overlap, due to the relatively low axial resolution when imaging at depth through tissue. When an ROI was initialized in each separate neuron, ABLE accurately detected the overlapping cell boundaries using the Euclidean distance dissimilarity metric (Eq. 2). On the Neurofinder Challenge (http://neurofinder.codeneuro.org/) dataset presented below (see Algorithm comparison on manually labeled dataset), ABLE demixed overlapping cells when performing segmentation with the correlation-based dissimilarity metric (Eq. 3; Fig. 3*G*).

### ABLE detects synchronously spiking, densely packed cells

**Figure 4.**
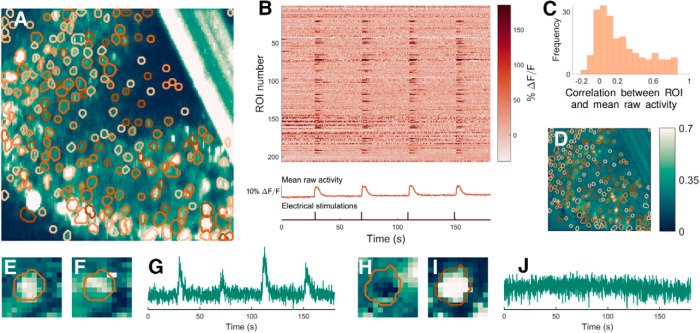
ABLE detects synchronously spiking, densely packed cells from mouse *in vitro* imaging data. The boundaries of the 207 detected ROIs are superimposed on the thresholded maximum intensity image (***A***) and the correlation image (***D***). For all correlation data we use Pearson’s correlation coefficient. ABLE detects ROIs that exhibit high correlation with the background (***C***) and neighboring synchronously spiking ROIs (***B***). ***B***, Neuropil-corrected extracted time courses of the 207 ROIs (each plotted as a row of the matrix) along with the video mean raw activity and the time points of the electrical stimulations. ***C***, Histogram of the correlation coefficient between the mean raw activity of the video and the extracted time series of each ROI. ABLE detected both active (***E****-****G***) and inactive ROIs (***H***, ***I***). We display the contours of the two detected ROIs on the correlation image (***E***, ***H***), the mean image (***F***, ***I***) and the corresponding extracted time courses (***G***, ***J***).

ABLE detected 207 ROIs from mouse *in vitro* imaging data (Fig. 4). Cells in this dataset exhibit activity that is highly correlated with other cells and the background as the brain slice was electrically stimulated (at rate 10 Hz for 2 s every 40 s) during imaging. When the cell interior and narrowband time courses are highly correlated, the external velocity of the active contour (Eq. 9) derived from the Euclidean distance dissimilarity metric (Eq. 2) is driven by the discrepancy between the baseline intensities of the subregions. This is evident when we consider the average time course of the cell interior (**f**^in^) and exterior (**f**^out^) as a sum of a stationary baseline component, the resting fluorescence, and an activity component that is zero when a neuron is inactive, such that $f^{\text{in}}\;=b^{\text{in}}+a^{\text{in}}$ and $f^{\text{out}}\;=b^{\text{out}}+a^{\text{out}}$. The time course of a pixel **x** is $I(x)=b^{x}+a^{x}$. Substituting these expressions into Equation 9, for pixels not in another cell, we obtain the external velocity $V(x)=||b^{x}-b^{\text{in}}|{|}^{2}-||b^{x}-b^{\text{out}}|{|}^{2}+R$, where the residual, *R*, encompasses all terms with contributions from the activity components. When the cell and the background are highly correlated, meaning that the discrepancy between activity components is low and, consequently, the contribution from *R* is comparatively small, the external velocity will drive the contour to include pixels with baselines more similar to the interior than the background. As a result of this, ABLE detected ROIs despite their high correlation with the background (Fig. 4*C*). Furthermore, inactive ROIs were detected (Fig. 4*H-J*), when their baseline fluorescence allowed them to be identified from the background (Fig. 4*I*).

The algorithm was automatically initialized on this dataset with 250 ROIs, initializations in the bar (an artifact that can be seen in the top right of Fig. 4*A*) were prohibited. Of the initialized ROIs, 19 were pruned automatically during the update phase of the algorithm as (1) their interior time course was not sufficiently different from that of the narrowband (3 ROIs), (2) they merged with another region (2 ROIs), or (3) they crossed the minimum and maximum size thresholds (14 ROIs).

### Algorithm comparison on manually labeled dataset

**Figure 5.**
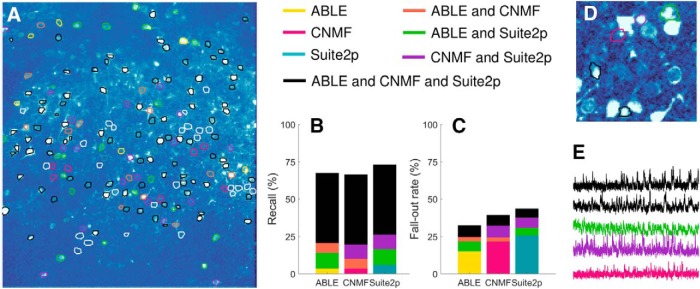
We compare the segmentation results of ABLE, CNMF (Pnevmatikakis et al., 2016), and Suite2p (Pachitariu et al., 2016) on a manually labeled dataset from the Neurofinder Challenge. On the correlation image, we plot the boundaries of the manually labeled cells color-coded by the combination of algorithms that detected them (***A***), undetected cells are indicated by a white contour. Suite2p detected the highest proportion of manually labeled cells (***B***), whereas ABLE had the lowest fall-out rate (***C***), which is the percentage of detected regions not present in the manual labels. Some algorithm-detected ROIs that were not present in the manual labels are detected by multiple algorithms (***D***) and have time courses which exhibit stereotypical calcium transient activity (***E***). The correlation image in ***D*** is thresholded to enhance visibility of local peaks in correlation. In ***E***, we plot the extracted time courses of the ROIs in ***D***.

We compared the performance of ABLE with two state of the art calcium imaging segmentation algorithms, constrained nonnegative matrix factorization (CNMF) (Pnevmatikakis et al., 2016) and Suite2p (Pachitariu et al., 2016), on a manually labeled dataset from the Neurofinder Challenge (Fig. 5). The dataset, which can be accessed at the Neurofinder Challenge website (http://neurofinder.codeneuro.org/), was recorded at 8Hz and generated using the genetically encoded calcium indicator GCaMP6s. Consequently, we apply ABLE with the correlation-based dissimilarity metric (Eq. 3), which is well suited to neurons with low baseline fluorescence and uneven brightness. As the dataset is large enough (512 × 512 × 8000 pixels) to present memory issues on a standard laptop, we run the patch-based implementation of CNMF, which processes spatially-overlapping patches of the dataset in parallel. We optimize the performance of each algorithm by selecting a range of values for each of a set of tuning parameters and generating segmentation results for all combinations of the parameter set. The results are visualized on the correlation image and the parameter set that presents the best match to the correlation image is selected. This process is representative of what a user may do in practice when applying an algorithm to a new dataset.

ABLE achieved the highest success rate (67.5%) when compared to the manual labels (Table 3). For a definition of the success rate and other performance metrics used, see Materials and Methods, Metric definitions. ABLE achieved a lower fall-out rate than Suite2p and CNMF (Fig. 5*C*), 67.5% of the ROIs it detected matched with the manually labeled cells. Some of the “false positives” were consistent among algorithms (Fig. 5*C*) and corresponded to local peaks in the correlation image (Fig. 5*D*), whose extracted time courses displayed stereotypical calcium transient activity (Fig. 5*E*). A subset of these ROIs may thus correspond to cells omitted by the manual operator. The highest proportion of the manually labeled cells were detected by Suite2p, which detected the greatest number of cells not detected by any other algorithm (Fig. 5*B*). A small proportion (13.2%) of cells were detected by none of the algorithms. As can be seen from Figure 5*A*, these do not correspond to peaks in the correlation image, and may reflect inactive cells detected by the manual operator.

**Table 3.** Algorithm success rate on manually labeled dataset

	Success rate (%)	Precision (%)	Recall (%)
ABLE	67.5	67.5	67.5
CNMF	63.4	60.7	66.5
Suite2p	63.7	56.5	73.1

On a manually labeled dataset from the Neurofinder Challenge, we compare the performance of three segmentation algorithms: ABLE, CNMF (Pnevmatikakis et al., 2016), and Suite2p (Pachitariu et al., 2016), using the manual labels as ground truth.

### Spikes are detected from ABLE-extracted time courses with high temporal precision

**Figure 6.**
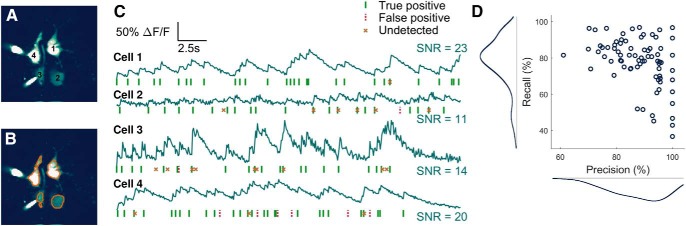
Spikes are detected from ABLE-extracted time courses with high accuracy. On an *in vitro* dataset (21 imaging videos, each 30 s long), we demonstrate spike detection performance compared to electrophysiological ground truth on time courses extracted from cells segmented by ABLE. We plot the labeled cells (***A***) and corresponding boundaries detected by ABLE (***B***) on the mean image of one imaging video. The extracted cellular time courses and detected spikes are plotted in ***C***. Spike detection was performed with an existing algorithm (Reynolds et al., 2016; Oñativia et al., 2013). On average over all videos, 78% of spikes are detected with a precision of 88% ***D***.

Typically, after cells have been identified in calcium imaging data, spiking activity is detected from the extracted cellular time courses and the relationship between cellular activity (and, if measured, external stimuli) is analyzed. On a mouse *in vitro* dataset (21 videos, each 30 s long), we demonstrated that time courses from cells automatically segmented by ABLE allow spikes to be detected accurately and with high temporal precision (Fig. 6). The dataset has simultaneous electrophysiological recordings from four cells (the electrodes can be seen in the mean image Fig. 6*A*), which enabled us to compare inferred spike times from the imaging data with the ground truth. We performed spike detection automatically with an existing algorithm (Reynolds et al., 2016; Oñativia et al., 2013). On average, over all cells and recordings, 78% of ground truth spikes are detected with a precision of 88% (Fig. 6*D*). The error in the location of detected spikes is less than one sample width, the average absolute error was 0.053 (s).

## Discussion

In this article, we present a novel approach to the problem of detecting cells from calcium imaging data. Our approach uses multiple coupled active contours to identify cell boundaries. The core assumption is that the local region around a single cell (Fig. 1*A*, inside the dashed box) can be well approximated by two subregions, the cell interior and exterior. The average time course of the respective subregions is used as a feature with which to classify pixels into either subregion. We assume that pixels in which multiple cells overlap have time courses that are well approximated by the sum of each cell’s time course. We form a cost function based on these assumptions that is minimized when the active contours are located at the true cell boundaries. Our results on real and simulated data indicate that this is a versatile and robust framework for segmenting calcium imaging data.

The cost function in our framework (Eq. 4) penalizes discrepancies between the time course of a pixel and the average time course of the subregion to which it belongs. To calculate this discrepancy, we use one of two dissimilarity metrics: one based on the correlation, which compares only patterns of temporal activity, the other based on the Euclidean distance, which implicitly takes into account both pattern and magnitude of temporal activity. When the latter metric is used, our cost function is closely related to that of Chan and Vese (2001). If we were to take as an input one frame of a video (or a 2D summary statistic such as the mean image), the external energy in our cost function for an isolated cell would be identical to the fitting term of Chan and Vese (2001). The lower-dimensional approach is, however, not sufficient for segmenting cells with neighbors that have similar baseline intensities. By incorporating temporal activity, we can accurately delineate the boundaries of neighboring cells (Fig. 3*A*).

We evolve one active contour for each cell identified in the initialization. Contours are evolved predominantly independently, with the exception of those within a few pixels of another active contour (see Materials and Methods, External velocity). In contrast to previous approaches to coupling active contours (Dufour et al., 2005; Zimmer and Olivo-Marin, 2005), we do not penalize overlap of contour interiors. This is because low axial resolution when imaging through scattering tissue can result in the signals of multiple cells being expressed in one pixel. We therefore permit interiors to overlap when the data are best fit by the sum of average interior time courses. Using this method, we can accurately demix the contribution of multiple cells from single pixels in real and simulated data (Fig. 3).

ABLE is a flexible method: we include no priors on a region’s morphology or stereotypical temporal activity. Due to this versatility, ABLE segmented cells with varying size, shape, baseline intensity and cell type from a mouse *in vivo* dataset (Fig. 2). Moreover, only two parameters need to be set by a user for a new dataset. These are the expected radius of a cell and *λ*, the relative strength of the external velocity compared to the regularizer (Eq. 5). To permit ABLE to segment irregular shapes such as cell bodies attached to dendritic branches (Fig. 2*A*), the weighting parameter, *λ*, must be set sufficiently high to counter the regularizer’s implicit bias toward smooth contours.

Unlike matrix factorization (Maruyama et al., 2014; Pnevmatikakis et al., 2016) and dictionary learning (Diego Andilla and Hamprecht, 2014), which fit a global model to an imaging video, our approach requires only local information to evolve a contour. To evolve an active contour, ABLE uses temporal activity from an area around that contour with size on the order of the radius of a cell. This allows us to omit from our model the spatial variation of the neuropil signal and baseline intensity inhomogeneities, which we assume to be constant on our scale. Our local approach means that the algorithm is readily parallelizable and, in the current implementation, runtime is virtually unaffected by video length (Table 1) and increases linearly with the number of cells.

Like any level set method, the performance of ABLE is bounded by the quality of the initialization, if no seed is placed in a neuron it will not be detected, if a seed is spread across multiple neurons they may be jointly segmented. In this work, we developed an automatic initialization algorithm that selects local peaks in the correlation and mean images as candidate ROIs. This approach, however, can lead to false negatives in dense clusters of cells in which the correlation image can appear smooth. In future work, an initialization based on temporal activity, rather than a 2D summary statistic, could overcome this issue. Our algorithm included minimal assumptions about the objects to be detected. To tailor ABLE to a specific data type (e.g., somas vs neurites), it is possible to incorporate terms relating to a region’s morphology or stereotypical temporal activity into the cost function. Furthermore, the level set method is straightforward to extend to higher dimensions (Dufour et al., 2005), which means our framework could be adapted to detect cells in light-sheet imaging data (Ahrens et al., 2013).

Here, we have presented a framework in which multiple coupled active contours detect the boundaries of cells from calcium imaging data. We have demonstrated the versatility of our framework, which includes no priors on a cell’s morphology or stereotypical temporal activity, on real *in vivo* imaging data. In this data, we are able to detect cells of various shapes, sizes, and types. We couple the active contours in a way that permits overlap when this best fits the data. This allows us to demix overlapping cells on real and simulated data, even in high noise scenarios. Our results on a diverse array of real datasets indicate that ours is a flexible and robust framework for segmenting calcium imaging data.

## Synthesis

Reviewing Editor: Leonard Maler, University of Ottawa Decisions are customarily a result of the Reviewing Editor and the peer reviewers coming together and discussing their recommendations until a consensus is reached. When revisions are invited, a fact-based synthesis statement explaining their decision and outlining what is needed to prepare a revision will be listed below. The following reviewer(s) agreed to reveal their identity: Jason Kerr

Both reviewers now agree that this Ms is substantially improved and suitable for publication in eNeuro. Although both reviewers have explicitly noted areas of concern that should be corrected, they also indicate that these corrections can be checked by the editor.

Below, I have given in full the essential points made by the reviewers. Please carefully address how you have edited the Ms in response to these reviews. I will then check your next iteration of the Ms. Please very carefully respond to all the very good technical points made by Reviewer#2.

Reviewer #1

Figure 3. Since the authors included a definition of SNR, it would be preferred

if the simulated data is expressed in terms of SNR, as opposed to \sigma values (0,100, 200).

Figure 3D, It would be best to plot the initialization contours against the correlation image so we can also see how the proposed initialization method works in practice.

Does the proposed method detect active but not silent neurons? This should be made explicit in the paper.

The regularizer R should be carefully defined and explained in an intuitive sense.

The comparison of ABLE with Suite2p and CNMF is welcome but the authors should be careful not to overstate their claims. For example, the big difference between precision and recall for CNMF indicates that the overall results could potentially be better with a different choice of

parameters (e.g., less strict acceptance criteria). The authors should make explicit how they used the other algorithms and whether they experimented with different parameter settings.

During the comparison the authors state that the size of the original video is prohibitively large. This is a confusing statement since the neurofinder datasets are not particularly large (< 10k frames which is equivalent to just a few minutes of recordings). The authors should make explicit how large of datasets their framework can handle. This is important since datasets keep getting larger and dealing with them efficiently is becoming increasingly important.

Also it is not true that CNMF cannot handle datasets of that size since the authors have published code that allows for processing of datasets up to 100k frames on single machines (see e.g., Giovannucci et al., Cosyne 2017). That makes downsampling down to 2Hz unnecessary and potentially harmful in terms of algorithm performance.

Finally, since the comparison will be publically available dataset it would be nice to state where one might access this dataset after publication of this Ms.

Reviewer #2

The authors have improved the manuscript by adding comparisons to existing methods, increasing the realism of their simulations and clarifiying some parts of the manuscript. The necessary analysis and description are now present in the paper but there are still some major shortcomings in both that need to be addressed.

Due to lack of detail and clarity in several places, the method is still not reproducible as described. There are also some technical but critically important issues remaining to be addressed with regard to how the simulations and comparisons to other methods were carried out.

1.The comparison to existing work is not convincing, and it is not at all clear that progress has actually been made here.

From the description in section 3.4, it's not at all clear that CNMF was used correctly, and there is almost no detail regarding how Suite2P was used. Why were data downsampled to 2HZ from CMNF but only to 6Hz for ABLE? What was done for Suite2P? This sort of analysis would clearly reduce CNMF's accuracy, as it used variation in time to separate different components, so averaging over time or other downsampling will reduce the information available to it. CNMF is capable of running on large fields of view acquired at 30 or 60 Hz, so it's not clear why this downsampling step would make sense here.

How were the different data chunks combined? If they were simply concatenated, this violates the models of signal and noise covariance built in to CNMF in an unnecessary way. If they were run separately, then 37.5 is simply too short a data segment, since many neurons with low firing rates may fail to fire a sufficient number of APs in that time.

Most in vivo experiments will produce at least 2 minutes of continuous data, if not much longer.

Which data from the Neurofinder challenge were used? What was the calcium indicator? What was the original imaging rate and image size in pixels? The precise recording(s) used should also be noted.

The way in which data were downsampled is not totally clear, but the discussion of boosting SNR suggests averaging. This should be clarified.

It's not clear what “prohibitively large” (line 28, p. 13) actually means.

If ABLE has a limit on the number or size of frames processed, this needs to be explicit stated.

Overall this analysis does not give the impression of a careful, unbiased evaluation.

2.The simulations are not adequately described. The simulation procedure should be described in such as way as to make it reproducible. In particular, what was the correlation between different simulated neurons? How does performance depend on this? This is an extremely important question when applying the algorithm in vivo, where conditions such as anesthesia or sensory stimulation may cause considerable synchronization of neighboring neurons.

How were the time series for each neuron generated before mixing them together in the simulation?

Was any background neuropil signal included in the simulations? Was it correlated to the neurons?

3.The manual annotations should be shown in Fig. 5. What was the criterion for defining nearby manual and automatic detections as matched? Some of the cells not detected by any of the automatic methods in Fig. 5 seem to show very high contrast from the background neuropil, so why weren't they detected?

4.Section 3.1: It's fine to say that there is no explicit prior on cell shape used, but clearly the regularization term in the cost function will induce some implicit prior. Since the regularization is never specified, it's hard to say what that may be. This should be reflected in the paper when priors/regularization are discussed.

5.p. 4: “For datasets in which the fluorescence expression level varies significantly throughout cells and, as a consequence, a pixel's resting fluorescence is not informative”. It's not at all clear how the second statement follows from the first.

6.“One of the advantages of the level set method is its natural capability of splitting and merging components” How is this an advantage, and as compared to what different method? Don't most/all available methods do this? Is there something about using level sets that makes this easier or more “natural”? If so, this should be explained.

Section 2.8 discusses merging based on “the noisy time courses.” Presumably, this refers to averages over the pixels interior to each of the two components which might or might not be part of the same cell. But shouldn't the expected SNR then depend on the size of each

component due to averaging over different numbers of pixels in different cases? For example, one would expect a higher correlation when dividing a neuron in half than when comparing 10% of its pixels to the other 90%. Shouldn't the threshold take this into account somehow?

The many combinations of different algorithms' outputs adds to the confusion in Fig. 5.

7.The the manuscript suffers from lack of clarity and important details are missing at several points: In section 2.1 the way the word “feature” is used is somewhat ambiguously. Maybe it's better to speak of a mathematical function defined on the the time courses of a set of pixels? If it's always going to be a space or time average in this paper, I would suggest using that definition instead of “Feature” or “function” for clarity, and then the possibility of using other functions can be mentioned in the discussion.

In equation 1 the notation is not quite clear. $\delta_\varepsilon$ is never clearly defined. Which approximation of the Heaveside function is used?

Section 2.5 is very difficult to understand as currently written. It's also not clear what is novel, what is standard practice, what is a precise result and what is an approximation. This might be help by creating a supplementary figure showing the various error terms and their

derivatives as images during the convergence process. What was the nature of the regulization term and what value of lambda was used? The paper is still very jargonheavy. For example, is it really necessary to refer to the objective function terms as “energy”? This is bound to confuse some readers without adding anything for those who do understand.

Section 2.6: “by a builtin MATLAB function” which function? What is the actual effect of alpha? Relative height as in relative to what? The technique needs to be described sufficiently well as to be reproducible.

Section 2.8 also does not describe how the contours are actually merged. What is done to combine their level set functions?

Section 2.9 RMSE measure is not clearly defined. RMSE of binned spike counts, spike times or something else?

The paragraph beginning on line 16 of page 11 seems to imply that a different background component is fit for different detected ROIs, but this is not clear.

The formula on line 14 in section 3.3 needs a more detailed derivation, as well as an explanation of why the formula is being considered and what it means for the algorithm in this context. The decomposition of f into a + b is not totally clear; e.g. is it assumed that a has a mean of zero.

## Author Response

Dear Editor,

We thank you and the anonymous reviewers for taking the time to review our paper. We have thoroughly revised the manuscript - we attach both a clean copy and a version in which all insertions and deletions are shown in blue and red shade, respectively. Large sections of text which have undergone substantial alteration are highlighted in the same blue shade as insertions. We include below a point by point response to each of the comments made by the reviewers. The page and line numbers refer to the clean copy of the manuscript.

Yours sincerely,

The Authors

Reviewer 1

1. Figure 3. Since the authors included a definition of SNR, it would be preferred if the simulated data is expressed in terms of SNR, as opposed to \sigma values (0,100, 200).

Each data-point in Fig. 3B corresponds to the success rate (at classifying pixels into cell interior or exterior) averaged over all cells in one realisation of a noisy video, where the noise has standard deviation sigma at all pixels. As the cells have different spike rates and baseline intensities, the SNR of each cell in the video is different. To display SNR versus average success rate, we would therefore have to calculate a summary statistic of the SNR over all cells. For this reason, we prefer not to display the results in terms of the SNR as this could be misleading and not directly comparable to, for example, the results in Fig. 5 where we display the SNR of time courses of single cells.

2. Figure 3D, It would be best to plot the initialization contours against the correlation image so we can also see how the proposed initialization method works in practice.

In the revised version of the paper on page 13 lines 2-3, we clarify that, although the proposed initialization algorithm is used on all real data examples in the paper, for the simulated data we initialize on a fixed grid (shown in Fig. 3D). This was initially stated only in the legend of the accompanying figure (Fig. 3), which we appreciate may not have been sufficiently clear.

We believe it is important to display the mean image in this figure to demonstrate that the cells in the video have differing baseline brightness levels and that ABLE can, despite this, delineate their boundaries accurately. As was emphasised by Reviewer 2 in the first round of review, this is a challenge often present in real data.

3. Does the proposed method detect active but not silent neurons? This should be made explicit in the paper.

Thank you for pointing out this omission, we agree it is important to make this point clearly. In the revised submission we demonstrate that, when the Euclidean distance cost function is used, ABLE can detect both active and silent neurons. We add 6 new subfigures (Fig. 4E - J) and some text (page 14 lines 2 to 3) to clarify this point.

4. The regularizer R should be carefully defined and explained in an intuitive sense.

We agree that detail on this was lacking in the previous manuscript. In the revised manuscript, we add an intuitive explanation (page 5 lines 22 to 25) of the regulariser, which we believe improves the clarity of the methods section. We believe that the full mathematical definition, which is lengthy and mathematically dense, is not appropriate to include in a manuscript in this journal. Although we do not state the full mathematical definition, we guarantee reproducibility by making our code available online (link in the manuscript on page 10 line 8) and referring readers to Li et al. (2010), for further detail.

5. The comparison of ABLE with Suite2p and CNMF is welcome but the authors should be careful not to overstate their claims. For example, the big difference between precision and recall for CNMF indicates that the overall results could potentially be better with a different choice of parameters (e.g., less strict acceptance criteria). The authors should make explicit how they used the other algorithms and whether they experimented with different parameter settings.

Thank you for pointing out this oversight, in the revised submission (page 14 lines 16 to 22) we have provided further detail on the implementation of CNMF and Suite2p and we discuss the method of choosing the parameters.

6. During the comparison the authors state that the size of the original video is prohibitively large. This is a confusing statement since the neurofinder datasets are not particularly large (< 10k frames which is equivalent to just a few minutes of recordings). The authors should make explicit how large of datasets their framework can handle. This is important since datasets keep getting larger and dealing with them efficiently is becoming increasingly important.

We agree that that statement could be misleading and so, in the revised manuscript on page 8 lines 7 to 13, we extend the discussion about the dataset size ABLE can handle. On page 8 lines 9-10, we highlight that ABLE's running time is only marginally affected by video length. ABLE is only limited by the memory of the MATLAB workspace - any video that can be loaded can be processed. As this is more of a workstation/implementational issue than an intrinsic algorithm issue, we do not delve into this point.

7. Also it is not true that CNMF cannot handle datasets of that size since the authors have published code that allows for processing of datasets up to 100k frames on single machines (see e.g., Giovannucci et al., Cosyne 2017). That makes downsampling down to 2Hz unnecessary and potentially harmful in terms of algorithm performance.

We thank the reviewer for pointing this out, in the revised manuscript we have used an updated version of CNMF that circumvents the memory issues presented by large datasets.

Consequently, we have been able to process all frames of the video rather than using contiguous chunks of frames. We feel this alteration has strengthened the manuscript.

In the initial submission, we performed downsampling (at rate 2Hz, thereby producing an imaging video with sampling frequency 4Hz) as a pre-processing step on the input dataset to CNMF as we found, on visual inspection, that it improved segmentation performance. Considering the comments from reviewer 1 and 2, we do not perform downsampling in the revised submission.

8. Finally, since the comparison will be publically available dataset it would be nice to state where one might access this dataset after publication of this Ms.

We agree that this would be beneficial. On page 14 line 13 of the revised manuscript we have indicated where the dataset can be found.

Reviewer 2

1) The comparison to existing work is not convincing, and it is not at all clear that progress has actually been made here.

a) From the description in section 3.4, it's not at all clear that CNMF was used correctly, and there is almost no detail regarding how Suite2P was used.

Thank you for pointing out this omission, in the revised submission (page 14 lines 16 to 22) we have provided further detail on the implementation of CNMF and Suite2p and we discuss the method of choosing the parameters.

b) Why were data downsampled to 2HZ from CMNF but only to 6Hz for ABLE? What was done for Suite2P? This sort of analysis would clearly reduce CNMF's accuracy, as it used variation in time to separate different components, so averaging over time or other downsampling will reduce the information available to it. CNMF is capable of running on large fields of view acquired at 30 or 60 Hz, so it's not clear why this downsampling step would make sense here. How were the different data chunks combined? If they were simply concatenated, this violates the models of signal and noise covariance built in to CNMF in an unnecessary way. If they were run separately, then 37.5 is simply too short a data segment, since many neurons with low firing rates may fail to fire a sufficient number of APs in that time. Most in vivo experiments will produce at least 2 minutes of continuous data, if not much longer. The way in which data were downsampled is not totally clear, but the discussion of boosting SNR suggests averaging. This should be clarified.

We thank the reviewer for pointing out the problem with running CNMF on contiguous data chunks. In the revised manuscript, we have used an updated version of CNMF that circumvents the memory issues presented by large datasets. Consequently, we have been able to process the entire video rather than using contiguous data chunks. We feel this alteration has strengthened the manuscript. We also take the reviewer's point that it is not

appropriate to downsample videos prior to applying CNMF, we do not perform downsampling in the revised submission.

c) Which data from the Neurofinder challenge were used? What was the calcium indicator? What was the original imaging rate and image size in pixels? The precise recording(s) used should also be noted.

We appreciate that it is helpful to add more detail about this dataset, in the revised submission (page 14 lines 12 to 16) we have provided further detail on the Neurofinder dataset and pointed to where it can be accessed online.

d) It's not clear what “prohibitively large” (line 28, p. 13) actually means. If ABLE has a limit the number or size of frames processed, this needs to be explicit stated. Overall this analysis does not give the impression of a careful, unbiased evaluation.

In the revised manuscript on page 8 lines 7 to 13, we extend the discussion about the dataset size ABLE can handle. On page 8 lines 9-10, we highlight that ABLE's running time is only marginally affected by video length. ABLE is only limited by the memory of the MATLAB workspace - any video that can be loaded can be processed. As this is more of a workstation/implementational issue than an intrinsic algorithm issue, we do not delve into this point.

2) The simulations are not adequately described. The simulation procedure should be described in such as way as to make it reproducible.

a) In particular, what was the correlation between different simulated neurons? How does performance depend on this? This is an extremely important question when applying the algorithm in vivo, where conditions such as anesthesia or sensory stimulation may cause considerable synchronization of neighboring neurons.

We agree that it is important for a segmentation algorithm to be able to segment neighbouring neurons which exhibit synchronous activity. For this reason, in Figure 3 and Section 3.3 we demonstrate ABLE's performance on real data from an electronically stimulated brain slice in which neighbouring neurons and the background have highly correlated activity. In this simulated example, considering the helpful suggestions of reviewer 2 in the first round of review, we demonstrate ABLE's performance in a different important scenario: segmenting overlapping neurons with varying inter- and intra- cellular brightness.

b) How were the time series for each neuron generated before mixing them together in the simulation? Was any background neuropil signal included in the simulations? Was it correlated to the neurons?

Thank you for pointing out the lack of detail on this point, in the revised version of the paper (page 9 line 23 to page 10 line 6), we describe the method of simulating the videos.

3)

a) The manual annotations should be shown in Fig. 5.

As is stated in the accompanying legend, we show the manual annotations in Fig. 5A colourcoded by the combination of algorithms that detected them.

b) What was the criterion for defining nearby manual and automatic detections as matched?

We appreciate that, in the results section, it would have been helpful to point the reader to the subsection of the methods in which the matching criterion is defined. In the revised submission, we do this on page 14, line 24.

c) Some of the cells not detected by any of the automatic methods in Fig. 5 seem to show very high contrast from the background neuropil, so why weren't they detected?

In Fig. 5A we plot only the true positives - the contours of the manually labelled cells colourcoded by the combination of algorithms that detected them. We agree that there appear to be further cells in Fig. 5A that are not labelled by the manual operator. In the revised submission on page 14 lines 28 to 29, we point out this disparity between the manual labels and the reality. To highlight this point, in Fig. 5D we plot only 'false positives' - ROIs that weren't detected by the manual operator but were detected by the algorithms. As such, there are also regions of high contrast in this subfigure (true positives) that are not encircled.

4) Section 3.1: It's fine to say that there is no explicit prior on cell shape used, but clearly the regularization term in the cost function will induce some implicit prior. Since the regularization is never specified, it's hard to say what that may be. This should be reflected in the paper when priors/regularization are discussed.

We appreciate that detail was lacking on this in the original manuscript, in the revised submission on page 5 lines 22 to 25, we provide an intuitive explanation of the regularizer, which we believe improves the clarity of the method. We also add a note in the discussion (page 16 lines 17 to 19) that the weighting of the data-based energy term and the regularizer needs to be done carefully, as the regularizer has an implicit bias towards smooth contours.

5) p. 4: “For datasets in which the fluorescence expression level varies significantly throughout cells and, as a consequence, a pixel's resting fluorescence is not informative”. It's not at all clear how the second statement follows from the first.

We take the reviewer's point that this sentence was initially unclear. In the revised submission on page 4 lines 13-15 we reformulate this sentence.

6) “One of the advantages of the level set method is its natural capability of splitting and merging components”

a) How is this an advantage, and as compared to what different method? Don't most/all available methods do this? Is there something about using level sets that makes this easier or more “natural”? If so, this should be explained.

We did not intend to imply that other segmentation algorithms cannot do this. We have removed this phrase from the manuscript.

b) Section 2.8 discusses merging based on “the noisy time courses.” Presumably, this refers to averages over the pixels interior to each of the two components which might or might not be part of the same cell. But shouldn't the expected SNR then depend on the size of each component due to averaging over different numbers of pixels in different cases? For example, one would expect a higher correlation when dividing a neuron in half than when comparing 10% of its pixels to the other 90%. Shouldn't the threshold take this into account somehow?

We agree that the strategy proposed by the reviewer is sensible, consequently we attempted this strategy on simulated data. However, we found that it did not introduce a sufficient advantage to justify the enhanced complexity.

7) The many combinations of different algorithms' outputs adds to the confusion in Fig. 5.

We appreciate that Figure 5 is not immediately easy to understand. We do, however, believe that it is informative to visualise the segmentation results in terms of the commonalities and differences in algorithm outputs. Having considered various options, we believe that this is the best choice.

8) The manuscript suffers from lack of clarity and important details are missing at several points.

a) In section 2.1 the way the word “feature” is used is somewhat ambiguously. Maybe it's better to speak of a mathematical function defined on the the time courses of a set of pixels? If it's always going to be a space or time average in this paper, I would suggest using that definition instead of “Feature” or “function” for clarity, and then the possibility of using other functions can be mentioned in the discussion.

We agree that the structure you suggest would increase the clarity of the description. Accordingly, we directly define the feature as the average subregion time course on page 3 lines 20-22.

b) In equation 1 the notation is not quite clear.

We have checked that all the terms in Eq. 1 have been defined prior to or alongside their usage in this equation.

c) $\delta_\varepsilon$ is never clearly defined. Which approximation of the Heaveside function is used?

We agree that some readers may want to know the mathematical definition of $\delta_\varepsilon$. As such, in the revised submission on page 6 line 18, we add a reference to two papers who used the same standard approximation and who state it fully. Due to the varied backgrounds of the readership of eNeuro we prefer not to add formulas where we do not believe they will be of general interest and add considerable value.

d) Section 2.5 is very difficult to understand as currently written.

We have re-written page 6 lines 15 to 22 to improve the intelligibility of this section.

e) It's also not clear what is novel, what is standard practice, what is a precise result and what is an approximation. This might be help by creating a supplementary figure showing the various error terms and their derivatives as images during the convergence process.

Thank you for pointing this out. We have re-written page 6 lines 15 to 22 referring to the methods that are standard practice and the terms that are approximations. Unfortunately, eNeuro do not accept supplementary information so we will not be able to provide the supplementary figure that you suggest.

f) What was the nature of the regulization term and what value of lambda was used?

See answer to Reviewer 1, Question 4 for changes to the manuscript with respect to discussion of the regularization term. In the revised submission, we specify the value of lambda used on each real dataset on page 6 line 13.

g) The paper is still very jargonheavy. For example, is it really necessary to refer to the objective function terms as “energy”? This is bound to confuse some readers without adding anything for those who do understand.

To be consistent with the level set literature, we prefer to retain the term `energy'. This is a term commonly used in many fields to refer to the objective function, so we hope readers will be familiar with its usage.

h) Section 2.6: “by a builtin MATLAB function” which function? What is the actual effect of alpha? Relative height as in relative to what? The technique needs to be described sufficiently well as to be reproducible.

Thank you for pointing out the lack of clarity here - we have added this detail on page 7 lines 23 to 27. The initialization code will also be provided in the online repository, guaranteeing reproducibility.

i) Section 2.8 also does not describe how the contours are actually merged. What is done to combine their level set functions?

On page 8 lines 21 to 25 we clarify how contours are merged.

j) Section 2.9 RMSE measure is not clearly defined. RMSE of binned spike counts, spike times or something else?

We have clarified this on page 9 lines 20 to 21.

k) The paragraph beginning on line 16 of page 11 seems to imply that a different background component is fit for different detected ROIs, but this is not clear.

We modify page 12 line 5 of the revised manuscript to clarify this. We add a reference to section 2.5 where it is explained that, in practise, a different background component is fit for each ROI.

l) The formula on line 14 in section 3.3 needs a more detailed derivation, as well as an explanation of why the formula is being considered and what it means for the algorithm in this context. The decomposition of f into a + b is not totally clear; e.g. is it assumed that a has a mean of zero.

In the revised submission on page 13 lines 25 to 26, we clarify that we assume that the activity component (a) is zero when a neuron is inactive.
